# The Triune Man

**Published:** 1897-05

**Authors:** J. Frank Thornton

**Affiliations:** Plum, Texas


For the Texas Medical Journal.

### THE TRIUNE MAN.

BY J. FRANK THORNTON, M. D., PLUM, TEXAS.

### (1) MIND, (2) MATTER AND (3) FORCE.

MIND is a general entity, specialized in man, individualized in particular man; matter is a general entity specialized in man, individualized in particular man; force is a general entity, specialized in man, individualized in particular man. The three combined constitute the triune man. Each was created by God and has continued to exist without accretion or decretion, often modified in form or manifestation. The doctrine of man's formation from the dust of the earth holds true here as in other doctrines—for every chemical element in his architecture has been found in the earth. The carbon, oxygen, hydrogen, nitrogen, sulphur, phosphorous, iron, manganese, sillicon and eight or nine elements known to chemists have been found in the body of man, have also been found in the earth, and are constant reminders of man's relationship to mother earth. These elements are not united in the human body as they are in the inorganic world, but their complete arrangement in proximate principles of the matter of the triune man as contrasted with the simple combinations in the compounds of the mineral and vegetable kingdoms is attributed to a special force known as denominative nerve force—(consideration). This brings us to the third entity of the triune man. Force is as much a separate and individual entity as matter and mind. Force is neither matter nor mind; it cannot be matter for it is invisible, neither can it be mind, because it exists and controls the movements of matter even where there are no faculties of the mind.

The functions of secretions, excretions, muscular contractions and other physiological processes are performed without the mind's influence.

The motive power which animates the secreting of the liver. The cells of the malphignian corpuscle of the convoluted tubule of the kidney. The ameboid cells of the entestinal ville which gobble up the emulsified fat and carry it into the lacteals and runs every machine in the human body is nerve force. The function of an organ ceases to move when the nerve supply is cut off, and hence it cannot work without this motive power. The flow of nerve force and the circulation of nerve force are terms which should come into more general use. Physiologist Yeo says the idea they convey and express should receive more attention in the future than in the past. Words are thoughts; words are theories; words are facts; man speaketh what his mind dictateth with the assistance of nerve force. Some times a new combination of words open up a new channel for observation, amounting to a discovery, as Harvey's discovery of the circulation of the blood, and it is hoped that the expression of nerve force may open up a new field for discoveries which will reward the explorer with a key to many of the difficult problems of physiology. The normal circulation of nerve force through the various organs and tissues is essential to health. The blood cannot circulate without the circulation of nerve force through the heart. And the muscular walls of the arteries, veins and endothelial cells of the capillaries without animated nerve force. Tissue change is as much dependent of this nerve force as the circulation of the blood. The blood might be floated to the tissues, but the cells could not appropriate and cast off the effete matter into the blood without the influence of nerve force circulating through the corpuscle and plasma of the blood and absorption and tissue change all await the circulation of nerve force.

Force is a general entity which has collected atoms into molecules; molecules into masses; masses into globules; globules into systems and systems into one grand universe. No new force has been added to the universe or generated by new methods. Light, heat and electricity are co-relations to one original force which sweeps the earth, moon and stars around the central sun and causes the peach to fall to the ground also. The force which holds together the molecules of carbon in the diamond is the same which fastens the earth to the sun and keeps the atoms to their places. In zinc sulphate cohesions the disturbance by friction of molecules of certain bodies and by shifting atoms of other bodies by chemical action causes electricity by molecular change taking place in the human body, nerve force is turned loose and flows to the centre, there it accumulates as in a storage battery, ready to be sent through the system. Nerve force has a co-relation to chemical and physical force—it flashes along the conducting fibres of the cerebo spinal and ganglionic system, furnishing motive power to the body and transmits messages to the mind. Force leaps through the clouds. This proves true when we see the lightning and hear the clashing thunder permeating the clouds. But we see nothing and hear nothing—it decomposes the carbon dioxide ($C O^2$) setting the oxygen free and fixing the carbon in the woody fibre. The force is first chemical and physical, and then vegetative, then animative or nervous—passing from one plane to another, the lower lifting down and bringing up the higher. Force opens the leaves of the violets and scatters the fragrance upon the air to be conveyed to the peripheral cells of the olfactory nerve, and thence to the brain, hence we have a sensation called smell. Force paints the violet and is conveyed to the peripheral cells of the optic nerve, and then to the brain, and then we have a sensation called sight. Force vibrates the maiden's voice and is conveyed over the aural ossicle to the peripheral cells of the auditory nerve, and thence to the brain, and we have a sensation called sound. Force liberated by pressure upon the cells of nerves of general sensibility and thence to the brain, and we have a sensation called touch. Force liberated by coming in contact with sugar with the peripheral cells gustatory nerve, and is conveyed to the brain, and then we have a sensation called taste. The peripheral cells and central cells determine the kind of sensation, the intervening fibres are only conductors as wires between a telegraph station. Physical and chemical force changed into nerve force and circulating through the brain bring about a molecular change. There is also a superior force capable of affecting a great change, this force is the mind.

Mind is the first entity in the triune, and I have reserved it for the last in discussion in order to use the other two as a ladder to climb up on and gain a better view. The mind of man is in contact with the mind of God; matter of man is in contact with material environment, and the force of man in contact with physical force. Mind existed before the heavens and earth, and is a great advisor of force and matter, and is pronounced Omnipotent God the fiat, let there be light, and is pronounced for the Creator to decree, let us make man our own image. From the time God made man mind has occupied the throne of human microism, sometimes at war, and then at peace, but never subjugated. How the mind is confined in the human body, how its boundaries are fixed, its shape—whether that of the body as a whole or only that of the cerebrum—whether the cerebrum is its seat or only its organ of manifestation to be used at will or never be used as the mind may elect, finally, whether the mind occupies any particular part of the body, is in the body, on the body or around the body, the fact remains that it constitutes the ruling power of the triune man during life and departs at death. The mind stimulates the development and improvement of the brain, enabling it better and better as a medium of thought, and the depth and number of the convolutions increases or the chemical composition improves.

The mind of the poet Newton is not different from a baby's, it is different in quality in which the brain acts, the mind is perfect, the brain is never.

Mind, matter and force with the brain form the triune man.

